# Multi-level barriers and facilitators to implementing evidence-based antipsychotics in the treatment of early-phase schizophrenia

**DOI:** 10.3389/frhs.2024.1385398

**Published:** 2024-10-14

**Authors:** Allison J. Carroll, Delbert G. Robinson, John M. Kane, Avram Kordon, Jennifer Bannon, Theresa L. Walunas, C. Hendricks Brown

**Affiliations:** ^1^ Department of Psychiatry and Behavioral Sciences and Center for Dissemination and Implementation Science, Northwestern University Feinberg School of Medicine, Chicago, IL, United States; ^2^ Institute of Behavioral Science, Feinstein Institutes for Medical Research, Northwell Health, Manhasset, NY, United States; ^3^ Departments of Psychiatry and of Molecular Medicine, The Donald and Barbara Zucker School of Medicine at Hofstra/Northwell, Hempstead, NY, United States; ^4^ Northwestern University Feinberg School of Medicine, Chicago, IL, United States; ^5^ Center for Health Information Partnerships, Northwestern University Feinberg School of Medicine, Chicago, IL, United States; ^6^ Department of Medicine and Center for Health Information Partnerships, Northwestern University Feinberg School of Medicine, Chicago, IL, United States

**Keywords:** consolidated framework for implementation research, patient perspective, schizophrenia, coordinated specialty care, stakeholder engagement, implementation determinants, antipsychotic medication

## Abstract

**Introduction:**

Long-acting injectable (LAI) antipsychotic medications and clozapine are effective yet underutilized medical therapies in early intervention services. The purpose of this study was to conduct a pre-implementation evaluation of contextual determinants of early intervention programs to implement innovations optimizing LAI antipsychotic and clozapine use within a shared decision-making model.

**Methods:**

Semi-structured interviews explored barriers and facilitators to implementing LAI antipsychotics and clozapine in early intervention services. Participants were: prescribers (*n* = 2), non-prescribing clinicians (*n* = 5), administrators (*n* = 3), clients (*n* = 3), and caregivers (*n* = 3). Interviews were structured and analyzed using the Consolidated Framework for Implementation Research (CFIR 2.0).

**Results:**

Participants were supportive of using LAI antipsychotics, despite barriers (e.g., transportation, insurance coverage), while most were unfamiliar with clozapine (Innovation). Critical incidents (e.g., COVID-19) did not interfere with implementation, while barriers included lack of performance measures; stigma affecting willingness to take medication; and clozapine considered to be a “last resort” (Outer Setting). Treatment culture was described as client-centered and collaborative, and most participants indicated LAI antipsychotic use was compatible with clinic workflows, but some were in need of resources (e.g., individuals trained to administer LAI antipsychotics; Inner Setting). Participants on the healthcare team expressed confidence in their roles. Family education and collaborative decision-making were recommended to improve client/family engagement (Individuals). Participants related the importance of tracking medication compliance, addressing client concerns, and providing prescribers with updated guidelines on evidence-based treatment (Implementation Process).

**Discussion:**

Results may guide implementation strategy selection for future programs seeking to optimize the use of LAI antipsychotics and clozapine for early-phase schizophrenia, when appropriate.

## 1 Introduction

Early intervention services (i.e., coordinated specialty care; CSC) for early-phase schizophrenia (i.e., within the first 5 years after first episode) improves positive and negative psychosis symptoms, increases treatment engagement, decreases the likelihood of psychiatric hospitalization, increases involvement in meaningful activities, and improves overall symptom severity (1, 2). A key component of early intervention programs includes selecting the psychopharmacological interventions that are tailored to the needs of each patient. Unfortunately, patients in the early stages of illness are often reluctant to take medication or take it sporadically. As a result, relapse and rehospitalization rates are quite high. Early relapses can be particularly impactful and efforts should be made to reduce risk (3).

Long-acting injectable (LAI) forms of antipsychotic medications have the same ingredients as a daily oral pill, but they are slowly released in the body over time (typically 3–6 months). Several widely used antipsychotics are available in LAI formulations, including risperidone, paliperidone, aripiprazole and olanzapine. LAI formulations are typically indicated to enhance adherence, reduce the risk of relapse, and/or ensure patients who appear to be unresponsive are receiving the medication (3). Use of LAI, compared to oral, antipsychotics is associated with greater medication adherence and reduced rates of psychiatric hospitalization (4–6). Clinical trials that emphasized staff training and patient education found nearly 90% acceptance rates of LAI antipsychotics by patients (7) yet prescription of LAI antipsychotics in real-world settings remain low (9%–15%) (8, 9).

For patients with treatment-resistant schizophrenia (i.e., patients who show lack of response to treatment with two antipsychotic medications in either oral or LAI formulation), clozapine is the only medication that has demonstrated efficacy and has regulatory approval (3, 10)—including for those with first-episode or early-phase schizophrenia (11, 12). However, clozapine is also underutilized, especially for early-phase schizophrenia (13–15).

A number of prior investigations have sought to understand how the knowledge, beliefs, attitudes, and experiences of patients, family members, and healthcare providers contribute to underutilization of these medications (14, 16–21). For example, behavioral health providers under-estimate the level of acceptance of LAI antipsychotics among their patients, anticipating that their patients will have negative attitudes, lack of education, and issues with access (e.g., transportation) (16). Regarding clozapine, many psychiatrists are reluctant to prescribe clozapine, and believe that their patients who are treated with clozapine are less satisfied with their treatment than patients treated with other antipsychotics (20, 21).

Considerably less attention has been paid to the non-individual-level barriers to implementing these effective medications in clinical practice. One systematic review of barriers to LAI antipsychotic use identified poor coverage by insurance plans; administrative costs associated with the medication itself, storage, and training; and lack of infrastructure to administer LAI antipsychotic injections outside of a usual setting (e.g., while on vacation, in primary care clinics). Administrative and structural barriers to clozapine use include the burden of blood monitoring (for both patients and clinicians), limited resources in the healthcare system (e.g., trained staff, transportation services), and lack of infrastructure for monitoring clozapine use in community settings (22).

Limitations of prior studies include lack of structured theories or frameworks to guide the work and a shortage of integration of multilevel perspectives. The use of appropriate frameworks significantly improves the efficiency and effectiveness of implementation of evidence-based practices (23). Frameworks put forth specific constructs to guide the implementation plan, assist in the interpretation of results, and provide a shared language between researchers, practitioners, and those with lived experience. The Consolidated Framework for Implementation Research (CFIR), and the updated CFIR 2.0 (24), is a widely used determinants framework that is used to identify and explain how barriers and facilitators of the context may affect implementation of an evidence-based practice (24, 25). This information can then be used to select strategies to overcome barriers and leverage facilitators for successful implementation.

The purpose of this study was to conduct a pre-implementation evaluation of the contextual determinants of LAI antipsychotics and clozapine use in early intervention programs. We used the CFIR 2.0 (24) to elucidate barriers and facilitators to implementing an algorithm to support shared decision-making around prescribing of LAI antipsychotics and clozapine, from the perspective of individuals representing multiple levels of implementation. These results will guide implementation strategy selection for future studies and programs seeking to optimize the presentation of LAI antipsychotics and clozapine for early-phase schizophrenia in a shared decision-making approach to implement these medications, when appropriate.

## 2 Materials and methods

### 2.1 Study overview

This study was based in the Early Psychosis Intervention Network (EPINET), which includes 8 regional “hubs” and over 100 clinics providing CSC to early-phase schizophrenia patients across 17 states. EPINET is a national learning health system that seeks to improve early psychosis care by conducting large-scale, practice-based research. This study was conducted to identify implementation determinants (barriers and facilitators) and strategies to implementing an algorithm and training to increase the reach and adoption of appropriate LAI antipsychotics and clozapine use for early-phase schizophrenia. Initial interviews were conducted in March-May 2023 and analysis was conducted from May-June 2023. We then added client interviews, conducted in September 2023 and analyzed in October 2023. All procedures were approved by Northwestern University's Institutional Review Board.

### 2.2 Participants and recruitment

We recruited five groups of constituents associated with early psychosis clinics: (1) Prescribing healthcare professionals (Prescribers), including psychiatrists, family medicine physicians, advanced nurse practitioners, family nurse practitioners; (2) Non-prescribing healthcare professionals (Non-prescribing clinicians), including mental health therapists, clinical social workers, and occupational therapists; (3) Organizational leaders and administrative staff (Administrators), including executive leadership, principal investigators, and program directors; (4) Individuals diagnosed with early-phase schizophrenia who received or were offered an LAI antipsychotic and/or clozapine (Clients); and (5) Relatives or partners of Clients (Caregivers). We aimed to recruit at least 2 and up to 5 participants per constituent group to ensure variability and reach saturation, which has been shown to be achieved with 9–17 interviews (26). Due to difficulty recruiting clients from EPINET clinics, we expanded recruitment efforts to include clients and caregivers from the Recovery from Early Psychosis Program (REPP) at Northwestern Medicine.

Study team members circulated a study announcement and flyer via email to EPINET hubs and the REPP clinic. A member of the research team joined clinic team meetings to provide study information and answer questions. Interested individuals then self-referred to the study. Participants provided verbal informed consent prior to initiating study activities and were given a $50 giftcard for participating. Our final sample included 2 prescribers, 5 non-prescribing clinicians, 3 administrators, 3 caregivers, and 3 clients who were recruited from sites in Michigan (3), Oregon (2), Oklahoma (2), Florida (5), and Illinois (4). No further demographics were collected to preserve the anonymity of participants.

### 2.3 Interviews

Interview guides were constructed using the CFIR 2.0 (24); the interview guides are available as Supplementary Material. Two to six questions were selected for each of the five CFIR domains (Innovation, Outer Setting, Inner Setting, Individuals, and Implementation Process), focusing on constructs that members of the research team (two implementation scientists and two psychiatrists/schizophrenia researchers) identified as the most representative of the barriers frequently seen in the treatment of schizophrenia (Table 1). Each interview guide was tailored for the constituent role and comprised 12–16 questions. Interviews were conducted using the Zoom videoconferencing platform, lasted approximately 45 min, and were audio/video recorded for analysis.

**Table 1:** Consolidated framework for Implementation Research (CFIR), version 2, domains and constructs asked of the included constituents.

CFIR 2.0 domain or construct	Constituent				
	Prescriber (*n* = 2)	Non-prescribing clinician (*n* = 5)	Administrator (*n* = 3)	Client (*n* = 3)	Caregiver (*n* = 3)
Innovation					
Complexity	x	x	x	x	x
Cost	x	x	x	x	x
Outer setting					
Critical incidents	x	x	x	x	x
External pressure: performance measurement pressure	x		x		
Local attitudes	x	x		x	x
Inner setting					
Structural characteristics	x		x		
Culture	x	x	x	x	x
Compatibility	x	x	x		
Relative priority			x	x	x
Available resources	x		x	x	x
Access to knowledge & information	x	x			
Individuals					
Innovation deliverers/leaders & recipients	x	x	x	x	x
Implementation process					
Teaming	x	x		x	x
Planning	x	x	x		
Engaging: innovation deliverers & recipients	x	x		x	x

### 2.4 Data analysis

Analysis of the interview data was performed using Rapid Turnaround Qualitative Analysis (27, 28). One extensively trained primary coder (AJC) conducted content analysis coding from the Zoom transcripts, which had been corrected and confirmed from the video recordings. A structured summary template was developed based on CFIR 2.0 (24). The results were then reviewed and confirmed with another member of the research team (AK) who had watched all of the interview recordings.

## 3 Results

The results are organized by the CFIR 2.0 domains and constructs. Due to a lack of familiarity with clozapine among many of the participants, most barriers and facilitators described herein relate primarily to LAI antipsychotics. Where applicable, disagreement between constituent groups is noted. See Table 2 for a summary and exemplar quotes.

**Table 2:** Summary of qualitative results by consolidated framework for implementation research (CFIR), version 2, domain and construct.

CFIR domain and construct	Summary of results (barrier vs. facilitator^a^)	Exemplar quotes (constituent)
Innovation		
Complexity	• Finding the right medication for each client can be difficult (barrier) • Some found LAI antipsychotic delivery to be largely straightforward while others found it to be more difficult (both) • Patient-level factors (e.g., schedule, transportation) (barrier) • Pharmacy coordination: transportation to pick up the LAI antipsychotic and bring the LAI antipsychotic to the clinic for the injection, noting that missing or delaying an injection can alter the client's medication schedule (barrier) • Insurance: coverage of LAI antipsychotics, un/under-insured clients, frequent changes in insurance coverage, insurance requirements (barrier) • Client perspective: fear of taking a shot, rotating injection sites, and inability to quickly adjust dosing (barrier)	• “The difficult part was figuring out what she had, what was going on, and how to address it. It took a long time to find the perfect cocktail of medications” (Caregiver) • “[LAI antipsychotic injection] goes rather smoothly. It's pretty quick. The one's who do it…they enjoy coming once a month” (Non-prescribing clinician) • “You gotta shake it, and you gotta do all this stuff. If you're not doing it all the time you can get out of practice” (Administrator) • “Money, insurance, knowledge. There's a lot of hurdles”. (Caregiver) • “Main problem is transportation barriers, getting people to be able to consistently engage in getting up here for med clinic”. (Non-prescribing clinician) • “Biggest barrier – and I've had it more than once – is with insurance companies not wanting to cover [LAI antipsychotics] unless they've tried at least two other medications…a lot of prior authorization and steps in the process”. (Prescriber) • “We'll try to do everything we can to get insurance to buy-in and get it approved, but sometimes that's also at the cost of [the patient] losing their stability…I will go get clients as long as they're willing and able and it's feasible. I have brought plenty of clients to their med appointments” (Non-prescribing clinician) • “Maybe it won't be as good because I could take the medicine twice per day versus the injectable might wear off” (Client)
Costs	• Financial costs to the system: paying someone to administer injections and/or perform blood draws (barrier) • Other costs to the system: designated space (both) • Financial costs to clients: out-of-pocket costs for medication, transportation (barrier) • Other costs to clients: undergoing blood draws, coordinating medication pick-up, time spent waiting to start a medication or being unable to continue an effective medication due to insurance hold ups, experiencing medication side effects (barrier)	• “Our Medical Assistants are the ones that are typically administering the LAI [antipsychotic]” (Administrator) • “Mainly just time…and having a space – at our clinic, space is a premium” (Administrator) • “Medication costs…Transportation costs…Insurance; We try to give them breaks, try to make sure they get insurance” (Non-prescribing clinician) • “The cost of having to wait to get that medication started because you're in the process of trying to get the insurance approval” (Prescriber) • “I currently have Medicaid so I don't pay for my medications” (Client) • “The cognitive effects…were pretty substantial – these would build with time, with the number of injections in, and there was just a point where…the medication wasn't fun” (Client)
Outer setting		
Critical incidents	• System impact: COVID and other critical incidents (e.g., inclement weather) do not significantly interfere with their ability to provide care to their clients, including LAI antipsychotic administration. (facilitator) • Client impact: those diagnosed with COVID, who did not want to wear a mask (barrier) • Mental toll on clients of living in a pandemic (barrier)	• “There's a window of time so I would say it's minimal that there'd be a disturbance on our end or logistics to getting [LAI antipsychotic administration] done…COVID didn't affect us too much. We really stayed open the whole time” (Administrator) • “We responded very proactively. We got telehealth services set up very quickly…We also started…mailing out medications” (Administrator) • “A lot of our clients – that is often a source of their paranoia at one point” (Non-prescribing clinician) • “Fear plays a big role because when they're in psychosis, the paranoia…they did not want to be in a crowd…fear of getting a disease” (Caregiver) • “It does [play a factor] a little bit because if you're taking pills you have to go to the pharmacy and get the pills from the clerks and everything like that. So that's another group of people that you have to interact with to get what you need” (Client)
External pressure	• No official policies or plans for performance measures related to LAI antipsychotic or clozapine use currently exist. (barrier) • Recommendations for benchmark measures included medication compliance, number of medications tried before initiating clozapine, and client outcomes such as (re)hospitalization, symptoms (e.g., psychosis, negative symptoms, executive function), and participation in meaningful activities. (both)	• “Getting a better measure of compliance with meds – pill counts… Looking at drug screens or looking at serum antipsychotic levels, having them bring their bottles in so you can do a pill count…making that part of routine visit”. (Prescriber) • “All of us including myself, should be looking at the rate of response…they are still quite symptomatic, and sometimes they have executive function that is not captured…Measurement-based care…maybe using a scale…people may have difficulty with thinking things clearly”. (Prescriber) • “We want to keep clients out of inpatient…One of our goals for clients is that they're participating in some sort of meaningful activity (volunteering, working, going to school)…We want to fully treat the psychotic symptoms – making sure that the voices are under control, that they're not hearing voices anymore or having auditory/visual hallucinations, they're not experiencing any more disorganized thought or speech, the negative symptoms have decreased as well”. (Administrator)
Local attitudes	• Mental health stigma (barriers): ◦ Societally: stigmatization in the media, “harder” diagnoses (e.g., schizophrenia) not often talked about ◦ Clients: do not like to accept the diagnosis of schizophrenia, which makes them less open to taking medications such as LAI antipsychotics and clozapine ◦ Clinicians: may be hesitant to diagnose schizophrenia due to concerns about stigma • Medication stigma (barriers): ◦ these medications are often viewed as a “last resort”, only used after failure to use or benefit from other medications ◦ Clients may feel as though they are “not normal” because they require medication, as well as the side effects they may experience (e.g., weight gain, mental slowing). • Countering stigma (facilitators): ◦ Young adult leadership council ◦ Peer support specialists ◦ Promoting community in the clinics ◦ Speaking about mental health with national organizations ◦ Providing education and support for clients, families and healthcare teams	• Mental health stigma: ◦ “Still some reluctance [among clinicians] to even diagnose with schizophrenia because of the concern about the stigma”. (Administrator) ◦ “Our culture in general is very uneducated about mental illness…older tropes that are still floating around…and plus when you have these horrible incidents…” (Caregiver) ◦ “A lot of our clients do not accept nor receive that they are [schizophrenic], and I think that's the hard part, trying to get them to understand their illness, and some of my clients believe that it's demonic influenced…I think it varies with different variables pertaining to how and what they would think of their circumstances/situation- if they're in an urban area…if you were an educated person…I think they can cope with it” (Caregiver) ◦ “I think that there's less of a stigma now because there's so much exposure to the challenges of what's going on in our society right now”. (Caregiver) ◦ “With the media and movies, I think it really portrays it in such a negative way that it makes it so hard for people to take that diagnosis and think that they can have a normal life”. (Non-prescribing clinician) ◦ ““…psychosis is almost like the cancer diagnosis of mental illness” (Non-prescribing clinician) ◦ “mental health is being recognized a lot more lately, but not much is being recognized about schizophrenia” (Non-prescribing clinician) ◦ “I didn't realize how normal [clients’] daily lives could be”. (Non-prescribing clinician) ◦ “I don't really see telling people that I have [this diagnosis] as a negative. If anything, it's kind of like an interesting fact about somebody…some normal people will see it as like it just is what it is type of deal, and it just proves this person is even more special. But then other people might actually look at it as “oh they have a weaker mind and they have to deal with being mentally ill”.” (Client) • Medication stigma: ◦ “I think there is still some belief that it's a “last resort” and only after failure of other medications” (Administrator) ◦ “people would be generally hesitant to support having somebody who's experiencing a first episode being started on clozapine”. (Administrator) ◦ “I know the biggest stigma that we have is how the medication makes them feel- the side effects…not knowing about your medication that you're getting, and so that puts that stigma there for them like just flat out not wanting it because they don't know about it”. (Non-prescribing clinician) ◦ “[with LAI antipsychotic] the [clients] don't have to have their medication around, or if they're with their friends it's like they don't have to take their medication, or just going on a vacation, or “I'm going to stay overnight with a friend”…they don't have to think about taking the pill” (Prescriber) ◦ “Some people might think that being mentally ill and being on medication affects your sharpness and makes you loopy and out of it…I dunno, there's some validity to it, like there's definitely some symptoms that come with taking medication” (Client) ◦ “the tone changed when I mentioned that I was going from [one medication] to [another] when I was talking to…the physicians aide…It seemed things changed when I mentioned the change…I think there is some stigma”. (Client) • Countering stigma: ◦ “We have a really wonderful young-adult leadership council that has put together a presentation for both our staff and for the community – it's made up of people who have been diagnosed with schizophrenia and have gone through our program, and they tell their own stories and try to break down some of those barriers…that's been the most effective”. (Administrator) ◦ “Well I think maybe [Client] is an anomaly because [Client] got very into this reduce the…eliminate the stigma. [Client] is very open about [their] illness, and very much wanting to…be an example that you can be on these medications and…still [live] a meaningful life”. (Caregiver) ◦ “having a peer support specialist on the teams interacting with people if they're open to that…is really helpful” (Prescriber) ◦ “If we can get any family member that's willing to be of support for the client we'll also try to encourage family therapy. But sometimes it's just very challenging because some people just don't have family support…the environment has a huge impact on your mental health (Non-prescribing clinician)
Inner setting		
Structural characteristics	• Physical infrastructure ◦ Clinics described appropriate office space designated for injections and/or blood draws (facilitator) • Work infrastructure ◦ Only one team member (typically a medical assistant) was trained and available to administer LAI antipsychotics or complete blood draws for clozapine monitoring (barrier) ◦ Recruiting outpatient nurses has been difficult since the start of the COVID-19 pandemic (because better paying inpatient positions are available) (barrier) ◦ Team nurses or co-located groups can step in to conduct injections or blood draws (facilitator) ◦ Prescribing clinicians require additional training or certification to prescribe clozapine (barrier)	• Physical infrastructure ◦ “We have a certain office where the nurse gives all the injections” (Administrator) • Work infrastructure ◦ “Not all medical assistants are trained to do injections… Logistically, just having enough people trained to do it, the more the better. Plus the more MAs [Medical Assistants] are trained in it could bring it up – remind the prescribers…They really are pretty key in knowing the patients, and so I think if they're trained to administer it, it would be on the forefront of their mind”. (Administrator) ◦ “…right now it's just staffing, and that it's really hard to recruit nurses especially for outpatient. When we have an inpatient [option] that's attached to us that pays a whole lot more – that's where the RNs tend to go”. (Administrator) ◦ “We have other team nurses…[if] the med clinic nurse isn't here at that time [they'll] step in and do that too…Coming in to get the medication when it's delivered to the agency is tough [for a client], so our team nurse will bring it out to her”. (Prescriber) ◦ “With Clozapine it requires an extra level of training to prescribe them, so only certain providers can prescribe that, typically our psychiatrist, and then I believe we might have one APRN [Advanced Practice Registered Nurse] who's able to”. (Administrator)
Culture	• Toward the clients (facilitators) ◦ Meeting clients where they are ◦ Respect for clients ◦ Patience with clients and families ◦ Supporting clients toward accountability and responsibility • Within the team (facilitators) ◦ Evidence-based ◦ Collaborative (shared decision-making model) ◦ Innovative ◦ Ambitious ◦ Curious ◦ Compassionate ◦ Resourceful	• Toward the clients ◦ “We are definitely super patient-centered. I mean, we go above and beyond”. (Administrator) ◦ “Level of respect that we give the clients no matter what they're going through”. (Non-prescribing clinician) ◦ “The other thing we sort of don't believe in is disability – everyone should be able to support themselves independently…we have them find some kind of part-time work”. (Prescriber) ◦ “at the hospital…they're just trying to kind of put the fire out” whereas, “here [at site], it's pretty much person-centered. You're not being told what you're going to take”. (Caregiver) ◦ “Clinic is made for reaching your goals and goal setting” (Client) ◦ “you get to make relationships and get positive affirmation and support from people that are just like you” (Client) ◦ “Safety and fairness…balancing stability and happiness” (Client) • Within the team ◦ “Health equity is very much a shared value among the team…Things that promote health equity is a much easier sell for the team”. (Administrator) ◦ “We try to be innovative, evidence-based, up to date with newest research, open-minded. Collaboration, I think, is really important too”. (Administrator) ◦ “A lot of our team specifically just has a passion for people and helping people” (Non-prescribing clinician) ◦ “Collaborative. Ambitious. We're also curious to learn more”. (Non-prescribing clinician) ◦ “We're very much on board…All of the team members with the shared decision-making model. So talking with people, presenting options – when it comes to the medications…I always offer the first-line recommended meds from research, and then I'll explain what that means and then talk about the option for an injectable…If [the clients] are like “no I'm not interested in that”, that's fine, we have other options”. (Prescriber)
Compatibility	• LAI antipsychotics and clozapine are well integrated into current workflows (facilitator) • The team attempts to set the client up with everything they might need, including appointments, injections, and pharmacy coordination; the high frequency of reminders may take away from other responsibilities. (facilitator) • The time it takes for the prescriber to explain the LAI antipsychotic to clients and families may disrupt clinic visits. (barrier) • Lab work is sent to be processed externally. (barrier)	• “it's really pretty fluid” (Prescriber) • “…[it] just flows as part of all of their services that they get, you know, ensuring that they are coming to their med clinics, and if they miss it, helping them get that rescheduled, and you know, just helping find out what's the barrier to get them there and continuing to work with them…” (Non-prescribing clinician) • “Biggest thing would be that education part when they come in for their initial psych evaluation, if an injectable is brought up by either the doctor or the family it should be explained – the benefits, pros and cons of the injection…more so than just offering the medication”. (Non-prescribing clinician) • “We used to have an internal [site] lab, but now they contract out. That's the only piece that customer service at the lab with [external company] is not as great as we'd like it to be”. (Administrator)
Relative priority	• LAI antipsychotics and clozapine initiatives may come second to fidelity reviews and insurance audits. (barrier) • Other clinic procedures may compete with LAI antipsychotic and clozapine initiatives. (barrier) • Clients reported that their medications and non-pharmacological interventions were equally important (both)	• “Just about to have our fidelity review…Chart reviews to make sure certain things are included in the progress notes…safety plans… I could see [LAI antipsychotics getting rolled into fidelity review]…I think it's totally possible and feasible”. (Administrator) • “Try to get them seen by a doctor within 14 days of the intake…We also do what's called an initial care plan when they first come in for a screening that allows them to receive services for 60 days…try to have their intake completed within 45 days”. (Administrator) • “It's a combination of the two [therapy/groups and medication]. They support one another. The groups help you reach social…it has its benefits – things for mental wellness” (Client) • “…goals and stability. They really want you to be productive throughout the day…Goal-setting is a big thing in [clinic] group, making sure you're stable and getting the connections that you need, finding individuals to hang out with, and problem-solving”. (Client)
Available resources	• Necessary resources: delivering the injection, including the person administering the injection, getting the medication to the clinic, and conducting the prior authorization process. (both) • Existing resources: the MAs/nurses who were trained to deliver the injections; refrigerators and storage bins for medications and needles; brochures explaining LAI antipsychotics; the patient assistance program; and other “tricks” for getting medications at an affordable cost. (facilitators) • “Nice-to-have” resources: on-site phlebotomist to complete blood draws and good relationships with pharmacies. (both) • Can be difficult to find evidence-based counseling services. (barrier)	• “Main thing is the resources for…administer[ing] the injection when it's due, resources to help pick up the medication if that's what's necessary and deliver it to the patient, resources to do the prior auth process”. (Prescriber) • “To have more people trained in administering the injections” (Administrator) • “Hardest thing was finding…We go to counseling services but they wanted to do talk therapy. They weren't really getting at the issue”. (Caregiver)
Access to knowledge and information	• Training and education: all participants reported receiving training/education, all new employees receive training/education when onboarding, and ongoing training/education occurred pharmaceutical representative presentations, pamphlets, in-service trainings, and regular team meetings. (facilitator) • Prescribers noted that they can further access training through professional organizations (e.g., American Psychiatric Association), though there is no structured continuing medical education for LAI antipsychotics or clozapine. (facilitator) • Both prescribers and non-prescribing clinicians believed that all team members lacked information about LAI antipsychotics and, in particular, clozapine. (barrier)	• “[Site] will have those same trainings themselves…So sometimes we're going to more trainings than most people would just because of the population we work with, just to be a little more knowledgeable and to be able to express to people what options they have”. (Non-prescribing clinician) • “We do have a drug rep who comes in for [medication]…She's helped teach us quite a bit about it…We get overloaded with stuff sometimes – she'll go through graphs and charts and give us all the information about age groups and demographics and just how well it works, and seeing the comparison between LAIs and orals [antipsychotics]”. (Non-prescribing clinician) • “I have access to the [American Psychiatric Association]… and the APA has SMIs – Serious Medical Illness Advisors, and I belong to NEI [Neuroscience Education Institute] Global which has lots of archived lectures…The two of us don't have a…structured way of renewal of [Continuing Medical Education] on [LAI antipsychotics or clozapine]”. (Prescriber) • “It would be helpful for [other team members] to learn a little about the antipsychotics and LAIs – they definitively seem to understand the benefits in terms of compliance and outcomes for with LAIs [antipsychotics] but not so much for clozapine” (Prescriber)
Individuals		
Implementation team members	• Team members were comfortable and confident in their roles [capability] (facilitator) • Prescribers have more “impact” on client's decisions regarding medication choices [opportunity] (facilitator) • Non-prescribing clinicians can spend more time with the clients, build rapport, share their own experience with mental health and treatment, and support the client throughout the process. [opportunity] (facilitator) • Non-prescribing team members were less familiar with the potential benefits of clozapine compared to other antipsychotics. (barrier) • Leadership support is present for LAI antipsychotics and clozapine (facilitator)	• “Yes everyone I believe [everyone is comfortable and confident in doing their role]. Everyone…from me taking them to the appointment for their injection, from the nurse being knowledgeable where they can have the injection at as far as the site, is comfortable. The doctor speaking to them, letting them know that they are receiving their injectable today”. (Non-prescribing clinician) • “We don't try to push one or the other. We just try to encourage medication period…we want them to feel like they have a choice, because that way they're buying into it”. (Non-prescribing clinician) • “We have the luxury…of spending more time with the clients and building the rapport” (Administrator) • “For me – I'm not usually the one who brings up the conversation but I'm the one to help nudge them, if I hear they're having some hesitancies towards it, I like to bring up the conversation and just try to see what's going on and why they have their thoughts on it”. (Non-prescribing clinician) • “Manager for behavioral health…is really into research so she's pro LAI [antipsychotic] and is pretty knowledgeable”. (Administrator) • “…be able to have those conversations in a way the client is going to be receptive to without them feeling like we're trying to force them to do it, that they have some empowered decision-making” (Administrator) • “I don't think [other team members] are informed much about the benefits of clozapine compared to the other antipsychotics” (Prescriber) • “The clinic makes it easy. There's many nurses who are trained in the intramuscular injection” (Caregiver)
Innovation recipients	• Some clients and families may be hesitant to put an unknown substance such as LAI antipsychotic into their bodies without being able to control it (and the side effects) (barrier) • Clients may not always have the appropriate knowledge of LAI antipsychotics (barrier) • Clients who have taken LAI antipsychotics were confident and comfortable with them (facilitator)	• “So at first the [clients and caregivers] are a little hesitant about putting something in their bodies” (Non-prescribing clinician) • “A few clients’ parents are trying to lower their dosages of medications because of the weight gain” (Non-prescribing clinician) • “I know one person recently got off of the injectable…They didn't want to be on the injectable, and I guess they got rid of all their medication…They didn't like the side effects” (Client) • “Once the decision is made to [start a medication], then it's easy” (Caregiver) • “For me personally, I am 100% confident in them…They're super easy, and it also feels like the medicine is there. It doesn't feel like it's not working like how the pills do…when you take pills you definitely feel the medicines working, but with the LAI [antipsychotics], it feels the same way like it feels like the medicine is in your system and everything. I don't know how it works. I know it's like small pellets that slowly dissolve in your system daily…That's pretty remarkable technology. I am 100% confident that they work”. (Client)
Implementation process		
Teaming	• Weekly team meetings (facilitator) • Constant communication between team members to ask questions, validate the treatment plan, and provide updates (facilitator) • Collaborations with co-located teams, where available and appropriate (facilitator) • Common goals and values (facilitator)	• “We meet as a treatment team every week…We keep communication on as much needed basis as possible. If we come across something and it's outside of treatment team then we send emails, we directly email the prescribers/the med providers, and we just try to keep a good open communication as much as needed…If there's something I don't know how to do or that I feel like maybe is getting too heavy for me, I feel like I have a great support system that I can reach out to. They're going to just support and validate me”. (Non-prescribing clinician) • “Every week in our team meeting, we'll review the people I'm scheduled to meet with during the upcoming week for who I'm scheduled to see for a psych eval or for medication reviews, and then they'll give updates about that individual…In between the team meetings, the [team members] will either send me an email or they'll stop by my office or they'll give me a phone call if there are any questions or collaboration needed”. (Prescriber) • “We are involved in their resilience training…We have a clinician, a case manager – the case manager usually helps the client in their livelihood, their well-being welfare…procuring information on how that person can get assistance in rent or food stamps or social security…helping [clients] with first-time episode of schizophrenia to live a productive life with the psychosis”. (Caregiver) • “The help desk, when you call in – they're good at getting messages to the doctors. The doctors are good at getting back to you pretty quickly, and those things seem like pretty standard operating procedure for most, but probably not as many groups really do as good as [site] does”. (Caregiver)
Planning	• Outcome measurement to include medication compliance, side effect reporting and measurement, and physician adherence to established guidelines for LAI antipsychotics and clozapine. (both) • Importance of client and family perspectives and buy-in. (both) • Successful implementation would include prescribers talking with every client about LAI antipsychotics and/or clozapine; documentation of patient progress (e.g., symptom improvement); improved functional outcomes for clients (e.g., quality of life, meaningful activities); and decreased stigma. (both)	• “Really interested in seeing the blood work, knowing if they have any particular medical problems, if there are any concerns about metabolic issues with weight gain and things like that…and then making sure that…we're consistently seeing what the A1c is and making sure that gets entered into REMs [Rare and Expensive Case Management]…And then monitoring after that would be monitoring for any side effects, monitoring for how they're responding to it”. (Prescriber) • “Functional outcomes for the patient – just in terms of quality of life, whether they're pursuing a vocation either school or work or meaningful activity, hospitalization or lack thereof, or emergency room visits…Symptoms and symptom management is actually sometimes less important to patients and families than functioning and staying out of the hospital”. (Administrator) • “Going beyond the [traditional measures]…Success is…well the symptoms are not that critical to me actually. Success is functioning, satisfaction with life. So a person might have some residual positive symptoms or even delusions but if they are enjoying their lives…achieve life goals – that means work, relationships, school, even though they have symptoms” (Prescriber) • “Helpful to address some of the barriers to clients not agreeing to LAI [antipsychotics]. For example, we have some clients that feel like the injection or the medicine is poison…Maybe providing information to the providers whether that's a therapist or case manager, about how to help alleviate some of those concerns. We have some clients that a have a fear of needles….Injections are becoming more common but I think there is a little bit of discomfort with being prescribed an oral medication vs an injection where it stays in your system the whole time…just because it's new compared to what clients are used to so maybe providing more information on that as well as some psychoeducation”. (Administrator) • “Patient perspectives and just kind of understanding what their thoughts are on this while process…more of a buy-in is patient perspectives versus the provider perspective. Both would go in well, but I feel like that would be more helpful to some clients knowing another client's perspective versus the prover's perspective”. (Non-prescribing clinician)
Engaging	• Innovation deliverers ◦ Most difficult to engage prescribers. (barrier) ◦ Make the intervention a support for the prescribers rather than another task for them to complete. (facilitator) • Innovation recipients ◦ Realistic and balanced presentation of information. (facilitator) ◦ Family education: health literacy-friendly materials, client success stories, and an emphasis on the freedom and lifestyle that clients gain when switching to an LAI antipsychotic (facilitator) ◦ Collaborative decision-making: not making declarative statements, encouraging clients to find the right mix for themselves, building rapport, and acknowledging that medications don't help everyone. (facilitator)	• Innovation deliverers ◦ “Making it so that it feels like a help versus additional work as much as possible”. (Administrator) ◦ “Making sure that the doctors know all the information about the client and that they fit the medication that they're needing”. (Non-prescribing clinician) ◦ “Having the opportunity to connect sometimes with other prescribers just to hear their experiences or what are their concerns or what are the things that they see are benefits”. (Prescriber) • Innovation recipients ◦ “Be up front about what to expect, kind of outline a directory off them eventually just because they're not always going to be pleasant. It just seems like now I wonder about being on LAI [antipsychotics], especially [medication 2], [medication 1] was pretty miserable…There are nasty side effects about LAI [antipsychotics] like at this point it currently comes with a hefty dose of fatigue. It's just not something you want to experience” (Client) ◦ “Education of working collaboratively, working with listening to them, and then getting input from the client, making it a kind of collaborative decision to try it…Put all your declaratives in a question – don't make declarative statements…Having a family meeting and just saying this is what this is”. (Caregiver) ◦ “Best way to do that would be when the patient comes in for their monthly check-up to meet with [prescriber]… have the family members or caregivers or whoever it may be, sit in the room at least for the beginning and just have them be part of the conversation so that way they know what's going on” (Client) ◦ “Having educational materials available for the patients too – so there's some external information that's really geared towards what patients and families are concerned about. Emphasizing that not needing to have to remember to take your medication and the freedom that provides…emphasizing the lifestyle help that can be”. (Administrator) ◦ having the families learn [motivational interviewing] and learn the same skills as clinicians so they are more patient and tolerant and more supportive rather than punitive when they are speaking with their loved ones”. (Prescriber)

The *Innovation* domain refers to specifics about pharmacologic treatment interventions (i.e., LAI antipsychotics and clozapine). The *Outer Setting* domain refers to relevant settings or conditions outside the clinic. The *Inner Setting* domain refers to the relevant settings or conditions in the clinic itself. The *Individuals* domain refers to the roles and characteristics of individuals involved in or affected by the implementation. The *Implementation Process* domain refers to the strategies and procedures needed to implement LAI antipsychotics and clozapine within the clinics.

Where responses differed between constituents, an exemplar quote from each group is presented. Where constituents were in agreement, an exemplar quote from one participant is presented.

^a^ A barrier indicates something that will impede implementation. A facilitator indicates something that will positively help with implementation. Both indicates that something may be a facilitator or a barrier, depending on the context and whether it is available.

### 3.1 General perceptions

All participants were supportive of using LAI antipsychotics; prescribers spoke to the literature recommending second-generation LAI antipsychotics as first-line treatment. Some participants noted that the LAI antipsychotics were underutilized. LAI antipsychotics (compared to oral medications) reportedly removed clients' daily reminder of illness and a potential method for suicide, however, clients expressed initial concerns about putting something foreign in their body. The importance of having the family involved and on-board with medication decisions was emphasized. Several participants described clozapine as a “last resort” largely due to side effects. Several participants described participating in prior research studies which increased use of these medications, but this effect atrophied after study completion.

### 3.2 Innovation

The Innovation domain refers to specifics about the pharmacologic treatment interventions (i.e., LAI antipsychotics and clozapine).

#### 3.2.1 Complexity

LAI antipsychotics and the day-to-day procedures (e.g., try an oral form first, ensure team member availability for injection) were viewed as relatively straightforward. The complexity of LAI antipsychotics lays in the coordination between the pharmacy and the clinic to obtain the medication and with insurance companies. Insurance coverage of these medications (especially LAI antipsychotics) varies widely; clients' insurance coverage changes; or complex insurance requirements (e.g., using a medical pharmacy). A prescriber stated, “Biggest barrier – and I’ve had it more than once – is with insurance companies not wanting to cover [LAI antipsychotics] unless they’ve tried at least two other medications…a lot of prior authorization and steps in the process”. Participants reported that the programs strive to manage these complexities where possible (e.g., procedures and personnel dedicated to prior authorizations, providing transportation), but high barriers to access persist (e.g., repeating paperwork).

#### 3.2.2 Cost

Financial costs to the system included employing trained personnel to deliver injections and/or perform blood draws—though, it was also noted that having a nurse or other team member in that role saved the prescribing clinician time. Additionally, injections and blood draws require designated clinic space. To clients, the financial costs included the medications itself (if un/der covered by insurance) and transportation. Non-financial costs to clients included time (e.g., coordinating with pharmacy, attending the clinic, waiting for insurance) and the physical consequences (e.g., pain of injections, blood draws, side effects). One client described their experience as, “The cognitive effects…were pretty substantial – these would build with time, with the number of injections in, and there was just a point where…the medication wasn't fun”.

### 3.3 Outer setting

The Outer Setting domain refers to relevant settings or conditions outside the clinic.

#### 3.3.1 Critical incidents

Largely, COVID and other critical incidents (e.g., inclement weather) do not significantly interfere with a program's ability to provide care to their clients, including LAI antipsychotic administration. COVID did impact clients individually where they could not visit the clinic if they were diagnosed with COVID or did not want to wear a mask when receiving services, as well as the mental toll of living in a pandemic.

#### 3.3.2 External pressure: performance measurement pressure

Per the administrators and prescribing clinicians, no official policies or plans for performance measures related to LAI antipsychotic or clozapine use currently exist. Rather, they recommended relevant benchmark measures [e.g., medication compliance, (re)hospitalization, and participation in meaningful activities]: “We want to keep clients out of inpatient…One of our goals for clients is that they’re participating in some sort of meaningful activity (volunteering, working, going to school)” (Administrator).

#### 3.3.3 Local attitudes

Clients described experiencing stigma associated with schizophrenia, the medications, and the side effects of the medications (e.g., weight gain, mental slowing), such as: “the tone changed when I mentioned that I was going from [one medication] to [another] when I was talking to…the physicians aide…I think there is some stigma”. Healthcare participants described the negative impact of stigma on the mental health and wellbeing of clients, resulting in clients having greater difficulty accepting a diagnosis of psychosis. Some also described a hesitance on the part of clinicians to diagnosis schizophrenia due to concerns about stigmatization. Regarding the medications themselves, healthcare participants described these medications (especially clozapine) as a “last resort”. In contrast, they noted LAI antipsychotics may reduce stigma by removing the daily reminder (when taking a pill) of mental health problems. Participants also described examples of countering stigma, such as asking clients to share their stories and by employing peer support specialists. As a prescriber noted, “having a peer support specialist on the teams interacting with people if they’re open to that…is really helpful”.

### 3.4 Inner setting

The Inner Setting domain refers to the relevant settings or conditions in the clinic itself.

#### 3.4.1 Structural characteristics

Per administrators and prescribing clinicians, ***physical infrastructure*** factors were not barriers to LAI antipsychotics or clozapine administration. The primary ***work infrastructure*** barrier was limited availability of trained personnel to administer LAI antipsychotics, complete blood draws for clozapine monitoring, and prescribe clozapine. One participant noted difficulty hiring outpatient nurses with appropriate skills. However, several participants described how team nurses or co-located groups provided assistance when needed: “We have other team nurses…[if] the med clinic nurse isn't here at that time, [they’ll] step in and do that too” (Prescriber).

#### 3.4.2 Culture

Several participants spontaneously described their teams as “patient-centered” with some specific examples including respect for clients while also guiding clients toward accountability and responsibility. Within their teams, participants described their culture with words such as collaborative (consistent with a shared-decision making model), compassionate, and resourceful.

#### 3.4.3 Compatibility

Most participants (administrators, non-prescribing clinicians, prescribing clinicians) expressed that LAI antipsychotic and clozapine use were already well integrated with the clinic workflows: “it's really pretty fluid” (Prescriber). The team assists clients with everything needed to facilitate LAI antipsychotic injections, including scheduling appointments, performing injections, and coordinating with pharmacies. Some barriers to compatibility included the time for prescribers to explain LAI antipsychotics to clients/families, sending frequent reminders for clients and their families, and sending lab work to be processed externally. As an administrator described, “We used to have an internal [site] lab, but now they contract out. That's the only piece that customer service at the lab with [external company] is not as great as we’d like it to be”.

#### 3.4.4 Relative priority

Administrators identified some potential competing initiatives in the clinics. For example, they identified regular fidelity reviews and insurance audits that occur a few times per year. They also described regular clinic procedures that may be prioritized, such as seeing the client within 14 days of intake or smoothly transitioning clients from hospitalization to outpatient. Clients reported that the program and clinicians focused equally on medications and non-pharmacological interventions (e.g., therapy, making connections, setting goals): “It's a combination of the two [therapy/groups and medication]. They support one another. The groups help you reach social…it has its benefits – things for mental wellness” (Client).

#### 3.4.5 Available resources

Existing resources that facilitated LAI antipsychotics and clozapine use included the MAs/nurses who were trained to deliver injections and complete blood draws; refrigerators and storage bins for medications and needles; brochures explaining LAI antipsychotics; patient assistance program; and “tricks” for getting medications at affordable cost. A prescriber stated, “Main thing is the resources for…administer[ing] the injection when it's due, resources to help pick up the medication if that's what's necessary and deliver it to the patient, resources to do the prior auth process”. One caregiver noted it was difficult to find evidence-based screening and counseling services to receive an accurate diagnosis and effective, holistic treatment.

#### 3.4.6 Access to knowledge and information

All healthcare participants reported baseline and ongoing training and education about pharmacologic treatment interventions. Many described receiving updated data and research results about LAI antipsychotics via pamphlets, in-service trainings, and team meetings. Prescribers noted they have access to training for both LAI antipsychotics and clozapine through professional organizations (e.g., American Psychiatric Association). Participants indicated that team members would benefit from further education: “It would be helpful for [other team members] to learn a little about the antipsychotics and LAIs–they definitively seem to understand the benefits in terms of compliance and outcomes for with LAIs [antipsychotics] but not so much for clozapine” (Prescriber).

### 3.5 Individuals

The Individuals domain refers to the roles and characteristics of individuals involved in or affected by the implementation.

#### 3.5.1 Implementation team members

Participants agreed that healthcare team members are comfortable and confident in their roles and have positive views of LAI antipsychotics. For example, prescribers have more influence on a client's decision about medication, while non-prescribing clinicians build rapport with clients via time spent and shared experiences. Several participants indicated that they respect their clients' autonomy, such as a non-prescribing clinician who said, “We don't try to push one or the other. We just try to encourage medication period…we want them to feel like they have a choice, because that way they're buying into it”. Non-prescribing team members were less familiar with the potential benefits of clozapine. Prescribers suggested further training and education would increase non-prescribers' capability and motivation to recommend and support clients on clozapine. Finally, participants indicated they had strong leadership support for LAI antipsychotics and clozapine, as well as new research to developed further evidence for their use.

#### 3.5.2 Innovation recipients

Non-client participants described hesitation among clients and their families about putting [medication] into their bodies, especially in the injectable form. Others noted that, oftentimes, clients were not aware of LAI antipsychotics as an option. Another consideration was that clients and their families occasionally try to use lower doses of medications due to side effects (e.g., weight gain), which is more difficult with an LAI antipsychotic compared to a daily pill. In contrast, caregivers and clients were agreeable to LAI antipsychotics: “I am 100% confident in them…They’re super easy, and it also feels like the medicine is there. It doesn't feel like it's not working like how the pills do…” (Client).

### 3.6 Implementation process

The Implementation process domain refers to the strategies and procedures needed to implement LAI antipsychotics and clozapine within the clinics.

#### 3.6.1 Teaming

Nearly all healthcare participants mentioned weekly team meetings as collaborative and helpful for team members to align expectations and present a united front to clients. They also noted that team members are in regular communication outside of team meetings, as needed. A non-prescribing clinician stated: “If there's something I don't know how to do or that I feel like maybe is getting too heavy for me, I feel like I have a great support system that I can reach out to. They’re going to just support and validate me”. One team noted they collaborate with the co-located integrated primary care team to share resources. Participants described common goals among members within their teams. Caregivers also described good collaboration with the healthcare team: “We are involved in their resilience training…We have a clinician, a case manager [who] helps the client in their livelihood…procuring information on how that person can get assistance in rent or food stamps or social security…helping [clients] with first-time episode of schizophrenia to live a productive life with the psychosis”.

#### 3.6.2 Planning

Relevant outcomes of LAI antipsychotic and clozapine use among participants included medication compliance, prescribers’ adherence to guidelines, and medication side effects. Successful implementation would be demonstrated by prescribers talking with every client about LAI antipsychotics/clozapine; documentation of client progress (e.g., symptom improvement); wellbeing outcomes for clients (e.g., quality of life, meaningful activities); and stigma. One administrator noted the importance of “Functional outcomes for the patient – just in terms of quality of life, whether they’re pursuing a vocation either school or work or meaningful activity, hospitalization or lack thereof, or emergency room visits…Symptoms and symptom management is actually sometimes less important to patients and families than functioning and staying out of the hospital”. It was noted that tracking these outcomes can increase use of LAI antipsychotics and clozapine. Many participants also described the importance of gathering client and family perspectives about LAI antipsychotics and clozapine to gauge their understanding, concerns, and buy-in.

#### 3.6.3 Engaging

To engage ***innovation deliverers,*** participants noted that it can be difficult to get prescribers on-board because they are overbooked and do not spend as much time with clients. Thus, any intervention implemented would need to support prescribers rather than adding to their workload: “Making it so that it feels like a help vs. additional work as much as possible” (Administrator; e.g., proactively providing the necessary information for prescribing). Recommendations to engage ***innovation recipients*** included family education (e.g., client success stories), collaborative decision-making (e.g., presenting a balanced perspective), and applying evidence-based counseling approaches (e.g., motivational interviewing, cognitive behavioral therapy). As stated by a client, “Be up front about what to expect, kind of outline a directory off them eventually just because they’re not always going to be pleasant.”

## 5 Discussion

The purpose of this study was to evaluate the contextual determinants of implementing an algorithm to optimize appropriate use of LAI antipsychotics and clozapine within a CSC program for early-phase schizophrenia. Results from these interviews with multilevel constituents will form the basis of a matrix used to select the most relevant implementation strategies to address the prevalent barriers and leverage the key facilitators in the implementation of LAI antipsychotics and clozapine.

The identified strengths of CSC programs that support the use of LAI antipsychotics and clozapine may be leveraged by these and other programs. First, nearly all participants described a collaborative culture with common goals and respect for client autonomy. Second, several participants confirmed high compatibility of medication procedures with current workflows. Third, healthcare team members, clients, and caregivers described healthy communication and engagement between all constituent groups.

Barriers reported across participants included the costs of these medications (financial and other), lack of external pressure (e.g., benchmarks) to encourage use and mark fidelity, and pervasive stigma about psychosis and treatment. Administrators, providers, and clients/caregivers alike described competing demands that kept them from prioritizing medications. Participants noted limited knowledge among non-prescribing clinicians, staff, and clients/families about LAI antipsychotics and clozapine, which is a known barrier to use of these medications (29, 30). Interestingly, several healthcare team participants overestimated the frequency of LAI antipsychotic and clozapine use. The predominant belief that everyone in the clinic supports the use of LAI antipsychotic use is at odds with the data that LAI antipsychotics are under-prescribed (8, 9). In fact, one administrator noted that a review of program data found they were not prescribing LAI antipsychotics as much as they thought. Additionally, most of the non-prescribing participants viewed clozapine as a “last resort”, despite research indicating that the majority of clients and their caregivers are more satisfied, stable, and adherent after clozapine is initiated (18, 19).

Several evidence-based implementation strategies may be employed to address these barriers. First, it is clear that appropriate training and education of healthcare teams, clients, and caregivers is needed. For example, virtual training holds great promise for cost-effective, scalable, and effective training for working with individuals with severe mental illness (31–33). Second, audit and feedback strategies, in which a systematic review of prescribing behaviors may be shared back to clinicians, may inform healthcare providers about their actual use (and missed opportunities) of LAI antipsychotics and clozapine and encourage greater adherence to guideline recommendations (34, 35). Finally, practice facilitation is a multi-level strategy designed to increase the uptake of effective clinical innovations and build organizational capacity through activities such as training and education, workflow review, practice reports and discussion, and developing tailored program materials (36, 37). Practice facilitation has been shown to be effective across healthcare settings to manage a range of clinical conditions (38, 39).

These results should be considered within the context of their limitations. First, we did not collect demographic information about our participants to preserve their anonymity. Second, the EPINET and REPP clinics represent a particular type of clinic. Thus, the identified determinants and proposed strategies may not be applicable to more general mental health clinics. Moreover, the selection of clinics was not random and may not be representative. Nonetheless, using the framework of the five CFIR 2.0 domains across multiple clinics likely allowed us to identify broader patterns that influence treatment of early-phase schizophrenia in similar settings.

This study identified determinants of implementing LAI antipsychotics and clozapine to treat early-phase schizophrenia in early intervention services clinics. These results expand prior work examining individual perspectives and opinions about these treatments to identify outer and inner setting confounders, as well as potential strategies to leverage facilitators and overcome barriers to optimize appropriate use of these evidence-based treatments.

## Data Availability

The datasets presented in this article are not readily available because we wish to protect the privacy and confidentiality of our participants given the sensitive and personal nature of the contents of the qualitative data. Requests to access the datasets should be directed to Dr. Allison Carroll, allison.carroll@northwestern.edu.
