# Supplementary material for: Multi-level barriers and facilitators to implementing evidence-based antipsychotics in the treatment of early-phase schizophrenia
Source: Front Health Serv. 2024 Oct 14;4:1385398. doi: 10.3389/frhs.2024.1385398 (PMC11513390; doi:10.3389/frhs.2024.1385398)
Supplement: Supplementary file 1 [file Datasheet1.pdf]

Supplementary Material for "Multi-level barriers and facilitators to implementing evidence-based antipsychotics in the treatment of early-phase schizophrenia"

Carroll AJ, Robinson DG, Kane JM, Kordon A, Bannon J, Walunas TL and Brown CH (2024). Front. Health Serv. 4:1385398. doi: 10.3389/frhs.2024.1385398

Semi-structured Interview Guide: Master

I will ask open-ended questions related to the treatment of schizophrenia. Specifically, I'm interested in your experience with long-acting injectables (LAIs) and clozapine for patients that are diagnosed with schizophrenia. We are developing a study that implements an algorithm to support clinicians in deciding if a patient with schizophrenia would benefit from these medications. Your perspective is important to solving this issue.

(The CFIR 2.0 constructs represented and constituent(s) who were asked each question are indicated in parentheses.)

1. Introduction (all)
    a. What hub/clinic are you associated with?
    b. Please tell me a little about your/your loved one's/your clinical experience with schizophrenia.
    c. Do you have a formal role with the hub/clinic?
    d. Do you have a role with your state's mental health services division?

INNOVATION

2. What, if anything, do you know about LAIs and clozapine? What are your perceptions related to the use of LAIs and clozapine for the treatment of early onset psychosis? (general; all)

3. How complicated is it to implement LAIs and clozapine in clinical practice? Why? (complexity; all)

4. What are the costs to implementing LAIs and Clozapine? (cost; all)

If there are barriers identified, solicit potential strategies to address them.

OUTER SETTING

5. How do/could large-scale or unanticipated events (like COVID) affect implementation of LAIs and clozapine? (critical incidents; all)

6. What quality or benchmarking metrics, or established service goals, would support implementation of LAIs and clozapine? (external pressure: performance measurement pressure; prescribers, administrators)

7. What is your perception about stigma associated with schizophrenia? What about the use of LAIs and Clozapine for treatment? (local attitudes/conditions; prescribers, non-prescribing clinicians, clients, caregivers)

If there are barriers identified, solicit potential strategies to address them.

INNER SETTING

8. What are the characteristics of your organization that support the implementation of LAIs and clozapine? Inhibit the implementation of LAIs and clozapine? (Structural characteristics; prescribers, administrators)
    a. (probe for layout and space, technology, tasks and responsibilities of the workforce)

9. What are the shared values, beliefs and norms at your organization that may affect (positively or negatively) the implementation of LAIs and clozapine? (Culture; all)
    a. (probe for equality, equity, recipients, deliverers, and learning)

10. How do LAIs and clozapine fit, or not, within the established workflows, systems, and processes of your organization? (compatibility; prescribers, non-prescribing clinicians, administrators)

11. What are other important initiatives currently occurring in your organization? How important is the implementation of LAIs and clozapine compared to those other initiatives? (relative priority; administrators, clients, caregivers)

12. What resources does your organization have to implement LAIs and clozapine? What else is needed? (available resources; prescribers, administrators, clients, caregivers)
    a. (probe for funding, space, and equipment)

13. How do you feel about the level of guidance or training available to you regarding LAIs and clozapine? (access to knowledge & information; prescribers, non-prescribing clinicians)

If there are barriers identified, solicit potential strategies to address them.

INDIVIDUALS

14. How do you and others in your organization feel about LAIs and clozapine being implemented in your organization? How confident, prepared, capable, available are you and the team to implement LAIs and clozapine in your organization? (Innovation leaders; all)
    a. probe for high-level leaders, mid-level leaders, opinion leaders, facilitators, leads, implementation team members

15. How do you and others you know feel about the LAIs and clozapine being delivered in your organization? How confident, prepared, capable, available are you and the team to use LAIs and clozapine? (Innovation recipients; all)

If there are barriers identified, solicit potential strategies to address them.

IMPLEMENTATION PROCESS

16. To what degree does the Coordinated Specialty Care (CSC) team join together, intentionally coordinating and collaborating on interdependent tasks, to implement LAIs and clozapine? (teaming; prescribers, non-prescribing clinicians, clients, caregivers)

17. What information needs to be collected in order to best inform implementation of LAIs and clozapine? (planning; prescribers, non-prescribing clinicians, administrators)
    a. (probe for delivers, recipients, and context)
    b. What are the specific steps, milestones, and outcomes that would indicate the implementation was a success?
    c. How would you measure those?

18. In your opinion, how can we best engage clinicians/prescribers in the implementation of LAIs and clozapine? (engaging: innovation deliverers; prescribers, non-prescribing clinicians, clients, caregivers)

19. In your opinion, how can we best engage patients and families/caregivers in this process? (engaging: innovation recipients; prescribers, non-prescribing clinicians, clients, caregivers)

If there are barriers identified, solicit potential strategies to address them.

STRATEGIES PROBE

What solutions would you propose for the barriers you've identified today?
